# Prediction of clinically significant prostate cancer with a multimodal MRI-based radiomics nomogram

**DOI:** 10.3389/fonc.2022.918830

**Published:** 2022-07-15

**Authors:** Guodong Jing, Pengyi Xing, Zhihui Li, Xiaolu Ma, Haidi Lu, Chengwei Shao, Yong Lu, Jianping Lu, Fu Shen

**Affiliations:** ^1^ Department of Radiology, Changhai Hospital, Shanghai, China; ^2^ Department of Radiology, 989th Hospital of the joint logistic support force of the Chinese People’s Liberation Army, Luoyang, China; ^3^ Department of Radiology, Ruijin Hospital Luwan Branch, Shanghai Jiaotong University School of Medicine, Shanghai, China; ^4^ Department of Radiology, Ruijin Hospital, Shanghai Jiaotong University School of Medicine, Shanghai, China

**Keywords:** magnetic resonance imaging, nomogram, radiomics, prostate cancer, clinically significant

## Abstract

**Objective:**

To develop and validate a multimodal MRI-based radiomics nomogram for predicting clinically significant prostate cancer (CS-PCa).

**Methods:**

Patients who underwent radical prostatectomy with pre-biopsy prostate MRI in three different centers were assessed retrospectively. Totally 141 and 60 cases were included in the training and test sets in cohort 1, respectively. Then, 66 and 122 cases were enrolled in cohorts 2 and 3, as external validation sets 1 and 2, respectively. Two different manual segmentation methods were established, including lesion segmentation and whole prostate segmentation on T2WI and DWI scans, respectively. Radiomics features were obtained from the different segmentation methods and selected to construct a radiomics signature. The final nomogram was employed for assessing CS-PCa, combining radiomics signature and PI-RADS. Diagnostic performance was determined by receiver operating characteristic (ROC) curve analysis, net reclassification improvement (NRI) and decision curve analysis (DCA).

**Results:**

Ten features associated with CS-PCa were selected from the model integrating whole prostate (T2WI) + lesion (DWI) for radiomics signature development. The nomogram that combined the radiomics signature with PI-RADS outperformed the subjective evaluation alone according to ROC analysis in all datasets (all *p*<0.05). NRI and DCA confirmed that the developed nomogram had an improved performance in predicting CS-PCa.

**Conclusions:**

The established nomogram combining a biparametric MRI-based radiomics signature and PI-RADS could be utilized for noninvasive and accurate prediction of CS-PCa.

## Introduction

Prostate cancer (PCa) was the second commonest male malignancy in 2020 around the world, causing great harm to the male genitourinary system (1, 2). The descriptive phrase “clinically significant” is broadly utilized for differentiating PCa that might result in morbidity and/or death from harmless PCa subtypes. Such differentiation is critical because “insignificant” PCa not causing harm is commonly encountered (2, 3). Overtreatment of insignificant PCa is considered an important limitation of prostate-specific antigen (PSA) testing.

The European Association of Urology (EAU)-European Association of Nuclear Medicine (EANM)-European Society for Radiotherapy and Oncology (ESTRO)-European Society of Urogenital Radiology (ESUR)-International Society of Geriatric Oncology (SIOG) guidelines (2020 version) for PCa summarized the newest data and advised active surveillance (AS) or watchful waiting (WW) in PCa cases showing a Gleason score (GS) < 7, while clinically significant prostate cancer (CS-PCa) patients with GS ≥ 7 should undergo timely treatment and intervention because of increased risk of progression and short overall survival in clinical practice (2). Therefore, accurately evaluating CS-PCa preoperatively is critical for predicting long-term prognosis and selecting therapeutic options, which would result in more personalized and effective treatments. However, clearly defining CS-PCa is difficult.

The currently applied standard practice of MRI-targeted and template biopsy shows low diagnostic inaccuracy (4, 5). The IP1-PROSTAGRAM trial showed higher detection of CS-PCa with MRI Prostate Imaging–Reporting and Data System (PI-RADS) > 2 in comparison with transrectal ultrasound-guided prostate (TRUS) biopsy (6). However, cancer detection rates (CDRs) are only 6% and 9% for PI-RADS 1 and PI-RADS 2, respectively (4); high-grade cancer may still be missed especially with previous MRI showing suspicious lesions. Patients and clinicians should recognize the considerable uncertainty about prediction (2).

Currently early individualized detection attracts increasing attention. With recent progress in high-throughput analytical tools, radiomics models integrating clinical parameters show overt advantages in generating critical data regarding tissue properties otherwise not detectable by the naked eye (7–13). Indeed, increasing evidence suggests that radiomics could be superior in GS prediction in PCa over routine imaging strategies (14–17). However, which sequence and segmentation method could yield higher clinical benefit have not been evaluated. Thus, a comparison of the predictive capacity of combinations of sequences and various segmentation approaches is urgently required to establish the best radiomics methodology. Therefore, this study aimed to develop a radiomics model considering multimodal MRI and evaluate its predictive potential in CS-PCa with external validation.

## Materials and methods

### Participants

The current retrospective trial had approval from the local Institutional Review Board (Committee on Ethics of Biomedicine, Changhai Hospital; Committee on Ethics of Biomedicine, Ruijin Hospital Luwan Branch; Committee on Ethics of Biomedicine, 989th Hospital of the joint logistic support force of the Chinese People’s Liberation Army).

Individuals who underwent radical prostatectomy with pre-biopsy prostate MRI were searched in the hospitals’ databases. Exclusion criteria were: (1) no histological confirmation of PCa with baseline MRI in our institutions (2) no PSA test within 8 weeks prior to baseline MRI; (3) a history of previous therapy for prostate cancer; (4) poor quality of MR images (such as susceptibility artifact); (4) time from baseline MRI to surgical procedure exceeding 12 weeks.

**Figure 1:**
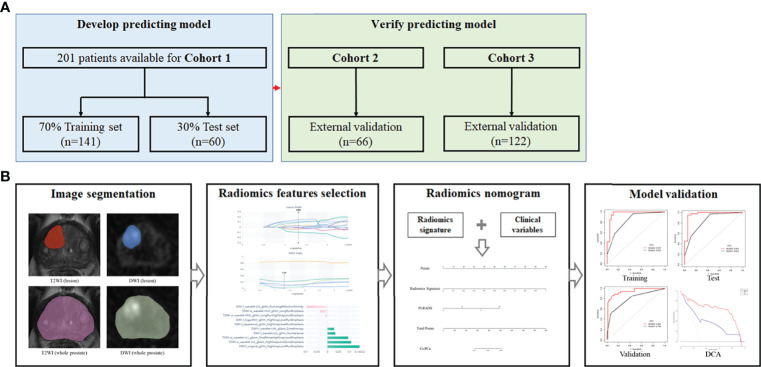
Study flowchart and nomogram workflow. **(A)** Study flowchart. Cohort 1, Changhai Hospital; Cohort 2, Ruijin Hospital Luwan Branch; Cohort 3, 989th Hospital of the joint logistic support force of the Chinese People’s Liberation Army. **(B)** Workflow for nomogram analysis.

Eventually, 201 cases were identified and enrolled in Changhai hospital from January 2016 to December 2019 as cohort 1. The primary cohort was randomized into the training set (n = 141) and test set (n = 60) at a ratio of 7:3. Next, 66 and 122 cases were enrolled from January 2019 to December 2021 in Ruijin Hospital Luwan Branch and 989th Hospital of the joint logistic support force of the Chinese People’s Liberation Army, respectively, as cohorts 2 and 3 (external validation sets), respectively. The study flowchart is shown in **Figure 1A**.

### Clinicopathologic data

Clinicopathology factors, including age, BMI, PSA levels, location of each tumor and GS post-prostatectomy, were retrieved from patient records. Radical prostatectomy samples underwent sectioning from apex to base at 3- to 5-mm intervals, and the PCa borders were delineated. All pathological GSs obtained from surgical samples were categorized as follows: GS < 7 [International Society of Urological Pathology (ISUP)] grade 1 PCa considered clinically insignificant; GS ≥ 7 (ISUP grade 2 and above) defined as clinically significant PCa (2, 3).

### Imaging and image analysis

Prostate MRI was carried out on a 3.0T MR scanner with an abdominal phase array coil without endorectal coil, following a 4-h fasting period and enema treatment with glycerin (20 ml).

Routine sequences, including sagittal T2WI, axial high-resolution T2WI, axial DWI, axial T1WI and gadolinium contrast-enhanced T1WI, were applied. **Supplementary Table 1** shows axial T2WI and DWI parameters utilized for PI-RADS and radiomics model development.

The PI-RADS (version 2.1) score for each case was assessed by three radiologists, including ZH.L., GD.J. and PY.X. with 8, 9 and 12 years of experience in MRI diagnosis, respectively, blinded to pathological data with the exception of tumor location. Any discrepancy among the three observers was resolved by discussion until at least two of them agreed.

### Image segmentation

The T2WI and DWI DICOM data acquired pre-biopsy were imported into the Radcloud radiomics platform (Huiying Medical Technology, China. http://radcloud.cn/). Since the original images were obtained from distinct cohorts, their normalization was critical to minimize signal variations for subsequent radiomics analysis (PyRadiomics package, class radiomics.imageoperations.normalizeImage; using the following formula: *f(x)=s(x−μ_x_)/σ_x_*, where *f*(*x*) indicates the normalized intensity; *x* indicates the original intensity; *μ* refers to the mean value; *σ* indicates the variance; *s* is an optional scaling, by default, it is set to 1. While reserving the diagnostic intensity discrepancy, the signal discrepancy in MR parameters was decreased). In addition, the resampling used (the radiomics.imageoperations.resampleImage function (the default interpolator is Bspline).

Two different segmentation methods were employed: (i) lesion segmentation, which only delineates the border that best fits the lesion area; (ii) whole prostate segmentation, which delineates the whole prostate region. Regions of interest (ROIs) were obtained by manual delineation in individual slices for each MR image (T2WI and DWI with b = 1500 s/mm^2^) by the above two segmentation methods in all specimens.

The first radiologist (GD.J.), who was blinded to clinical data, independently carried out the segmentation process for every case on the platform, comprising lesion segmentation and whole prostate segmentation, respectively. Then, ROIs were utilized to obtain volumes of interest (VOIs) in all cohorts. Next, two radiologists (ZH.L. and GD.J.) repeated segmentations for 30 random cases one week later for observer’s agreement analysis. Additionally, segmentations were performed under the supervision of a senior radiologist (F.S.), with 14 years of related work experience, for avoiding overt lesion misidentification.

### Radiomics feature extraction and reduction

Based on the derived VOIs, four groups of features were obtained: (1) **first-order** features, quantifying voxel intensity distribution on MR scans; (2) shape features, reflecting the 3D features of VOIs; (3) texture features, quantification of region heterogeneity differences, including gray-level co-occurrence, run length, size zone and neighborhood gray-tone difference matrices; (4) higher-order features, encompassing transformed first-order statistics and texture features, including logarithm, exponential, gradient, square, square root, local binary pattern [LBP] and wavelet transformations. In all, 1409 radiomics features were respectively obtained with the above platform from each VOI, based on the Python software package “pyradiomics” (version 6.1). Features complied with the image biomarker standardisation initiative (IBSI) standard (18).

For each cohort, inter- and intra-observer correlation coefficients (ICCs) were determined to assess feature robustness. Features with ICCs above 0.9 were subsequently utilized for model building, with excellent feature reproducibility.

Based on the two different segmentation methods, ten types of models were obtained: Model 1, DWI (lesion + whole prostate); Model 2, DWI (lesion); Model 3, DWI (whole prostate); Model 4, T2WI (lesion + whole prostate); Model 5, T2WI (lesion); Model 6, T2WI (whole prostate); Model 7, lesion (DWI + T2WI); Model 8, whole prostate (DWI + T2WI); Model 9, whole prostate (DWI) + lesion (T2WI); Model 10, whole prostate (T2WI) + lesion (DWI). For selecting optimal features related to CS-PCa in each model, the variance threshold algorithm, Select-K-best and the least absolute shrinkage and selection operator (LASSO) algorithm were employed.

### Radiomics signature building

The selected features (non-zero coefficients in the LASSO algorithm) were employed to develop a radiomics signature for scoring patients in the 10 models, respectively. The predictive value of the radiomics signature was assessed by determining the area under the receiver operator characteristic (ROC) curve (AUC) and Delong test in the training set.

### Nomogram model establishment

The predictive abilities of clinical variables and the radiomics signature were assessed by univariate logistic regression analysis. Parameters with *p*<0.05 were subsequently combined to build the nomogram model by multivariable logistic regression analysis (*p*<0.05). Next, the nomogram was examined for performance in each cohort. **Figure 1B** **shows** the nomogram’s workflow.

### Statistical analysis

The distribution of continuous data was evaluated by the Kolmogorov-Smirnov test, and the t-test or Wilcoxon test was utilized for comparing these data. The Chi-square or Fisher’s exact test was performed for qualitative data analysis. In the variance threshold approach, a threshold of 0.8 was applied, so that the eigenvalues of the variance smaller than 0.8 were removed. The select-K-best approach, which belongs to a single variable feature selection method, retained all features showing *p*<0.05. In the LASSO model, the L1 regularizer constituted the cost function, applying 5 as the cross-validation error and 1000 iterations at most (11–13). Sensitivity, specificity, accuracy, positive predictive value (PPV), negative predictive value (NPV), positive likelihood ratio (PLR) and negative likelihood ratio (NLR) were determined. The goodness of fit for the monogram was assessed by the Hosmer-Lemeshow test. AUC calculation, NRI, and the DeLong test were carried out for comparing the nomogram and PI-RADS V2.1. DCA was carried out for determining the nomogram’s clinical usefulness by assessing net benefits at distinct threshold probabilities. The nomogram was examined with R 3.6.3. The remaining data were assessed with SPSS (version 22.0, Inc., Chicago, IL, USA) and MedCalc v19.6.1. *P*<0.05 was deemed statistically significant.

## Results

### Patient features

**Table 1** lists the features of all patients. Clinicopathological parameters were similar in the three cohorts (*p*>0.05). According to pathological GS based on final surgical specimens, 139/201 (69.2%), 45/66 (68.2%) and 86/122 (70.5%) cases were defined as CS-PCa (GS ≥7) in the three cohorts, respectively.

**Table 1:** Clinical characteristics of patients with prostate cancer in all cohorts.

Characteristic		Cohort 1	Cohort 2	Cohort 3	*P* value
		(n=201)	(n = 66)	(n = 122)	
Age (year, mean ± SD)		58.547 ± 10.351	59.167 ± 10.181	58.492 ± 10.811	0.902
BMI (kg/m^2^, mean ± SD)		23.977 ± 2.706	23.664 ± 2.734	24.442 ± 2.971	0.153
Tumor location (%)	Peripheral zone	99 (49.3)	30 (45.5)	61 (50.0)	0.975
	Transitional zone	63 (31.3)	23 (34.8)	39 (32.0)	
	Peripheral + Transitional zone	39 (19.4)	13 (19.7)	22 (18.0)	
PI-RADS (%)	1	0 (0)	0 (0)	0 (0)	0.957
	2	60 (29.9)	16 (24.2)	36 (29.5)	
	3	34 (16.9)	14 (21.2)	24 (19.7)	
	4	75 (37.3)	24 (36.4)	42 (34.4)	
	5	32 (15.9)	12 (18.2)	20 (16.4)	
Gleason score (%)	<7	62 (30.8)	21 (31.8)	36 (29.5)	0.826
	7 (3 + 4)	48 (23.9)	15 (22.7)	24 (19.7)	
	7 (4 + 3)	42 (20.9)	12 (18.2)	25 (20.5)	
	8 (4 + 4 or 3 + 5 or 5 + 3)	38 (18.9)	11 (16.7)	24 (19.7)	
	9, 10	11 (5.5)	7 (10.6)	13 (10.6)	
Pathological T stage ^#^	T2	136 (67.7)	36 (54.5)	66 (54.1)	0.070
	T3a	34 (16.9)	17 (25.8)	35 (28.7)	
	T3b	31 (15.4)	13 (19.7)	21 (17.2)	
PSA (ng/ml, median IQR) ^*^		12.600 (7.782, 23.280)	12.525 (7.730, 20.578)	13.485 (9.479, 26.995)	0.493

Cohort 1: Training and test sets; Cohort 2: Validation set 1; Cohort 3: Validation set 2.

BMI: Body mass index; PI-RADS: Prostate imaging reporting and data system; PSA: Prostate-specific antigen; IQR: interquartile range.

^#^The current Union for International Cancer Control (UICC) no longer recognizes pT2 substages.

^*^Postoperative blood samples.

### Radiomics features

Feature repeatability based on ICCs in distinct cohorts is shown in **Supplementary Figure 1**. After inter/intraobserver agreement analysis, 1239/1409 T2WI (lesion) (87.9%), 1243/1409 T2WI (whole) (88.2%), 1096/1409 DWI (lesion) (77.8%) and 1199/1409 DWI (whole) (85.1%) features had excellent robustness and were subsequently utilized in radiomics analysis (inter- and intra-observer ICCs ≥0.9). There was excellent reproducibility for VOI size of lesion segmentation (ICC of T2WI, 0.931; ICC of DWI, 0.910) and whole prostate segmentation (ICC of T2WI, 0.942; ICC of DWI, 0.913). Eventually, optimal features were obtained with the LASSO algorithm for each model and presented in **Supplementary Table 2**.

### ROC analyses of the radiomics signature

**Figure 2:**
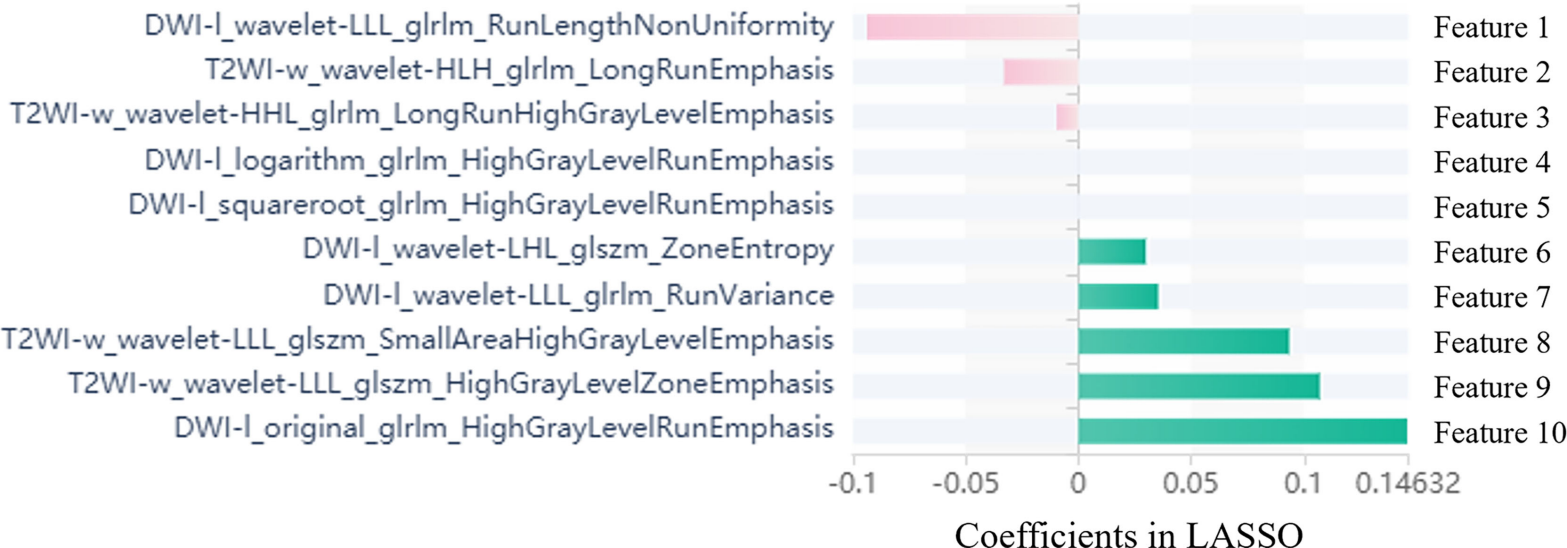
Selected radiomics features with associated coefficients in the LASSO model. DWI-l: lesion segmentation of DWI; T2WI-w: whole prostate segmentation of T2WI. GLSZM: Gray level size zone matrix; GLDM: Gray Level dependence; GLRLM: Gray level run length matrix; NGTDM: Neighborhood gray tone difference matrix; Wavelet: The wavelet transform decomposes the tumor area image into low-frequency components (L) or high-frequency components (H) in the x, y, and z axes.

The selected features were utilized for the radiomics signature (RS) in each model, respectively. The detailed ROC curve analyses for the 10 models, PSA and PI-RADS are listed in **Table 2**. ROC curves and their comparisons (Delong test) are shown in **Supplementary Figure 2**. Among the 10 models, PSA and PI-RADS, whole prostate (T2WI) + lesion (DWI) was determined to have the best performance by ROC curve analysis in the training set (AUC=0.967, specificity=90.9%, sensitivity=92.8% and accuracy=92.2%). The ten optimal features of whole prostate (T2WI) + lesion (DWI) are shown in **Figure 2**. The correlation analysis of selected features is shown in **Supplementary Figure 3**.

**Table 2:** ROC curve analysis in the training set.

	AUC	95% CI	Specificity	Sensitivity	Accuracy	PLR	NLR	PPV	NPV
**Model 10**	0.967	0.939-0.995	0.909	0.928	0.922	10.206	0.079	0.957	0.851
**Model 6**	0.929	0.883-0.976	0.841	0.948	0.915	5.962	0.061	0.929	0.881
**Model 8**	0.920	0.876-0.963	1.000	0.845	0.894	infinity	0.155	1.000	0.746
**Model 4**	0.911	0.862-0.960	0.704	1.000	0.908	3.385	0.000	0.882	1.000
**Model 3**	0.909	0.864-0.954	1.000	0.742	0.823	infinity	0.258	1.000	0.638
**Model 7**	0.903	0.854-0.952	1.000	0.722	0.808	infinity	0.278	1.000	0.620
**Model 9**	0.899	0.822-0.976	0.864	1.000	0.957	7.333	0.000	0.942	1.000
**Model 2**	0.888	0.836-0.941	0.886	0.784	0.816	6.895	0.244	0.938	0.650
**Model 1**	0.837	0.750-0.923	0.727	0.928	0.865	3.402	0.099	0.882	0.820
**PI-RADS**	0.835	0.766-0.904	0.545	0.969	0.837	2.132	0.057	0.825	0.889
**Model 5**	0.800	0.707-0.892	0.704	0.866	0.816	2.931	0.190	0.866	0.704
**PSA**	0.776	0.702-0.851	1.000	0.557	0.695	infinity	0.443	1.000	0.506

Model 1: DWI (lesion + whole prostate).

Model 2: DWI (lesion).

Model 3: DWI (whole prostate).

Model 4: T2WI (lesion + whole prostate).

Model 5: T2WI (lesion).

Model 6: T2WI (whole prostate).

Model 7: lesion (DWI + T2WI).

Model 8: whole prostate (DWI + T2WI).

Model 9: whole prostate (DWI) + lesion (T2WI).

Model 10: whole prostate (T2WI) + lesion (DWI).

AUC, area under the curve; PLR, positive likelihood ratio; NLR, negative likelihood ratio; NPV, negative predictive value; PPV, positive predictive value.

### Logistic regression analysis and nomogram model establishment

**Figure 3:**
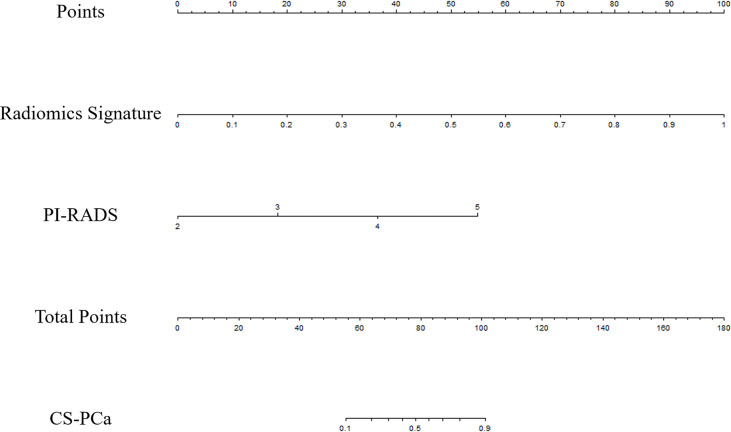
The nomogram developed using the training set for predicting CS-PCa, based on the radiomics signature and PI-RADS.

Univariate analysis showed the RS, PSA and PI-RADS had significant associations with CS-PCa. Then, predictive model development employed multivariate logistic regression analysis of the selected risk factors (PI-RADS, OR=7.688, *p*=0.011; RS, OR=7.650×10^5^, *p=*0.002) in the training set (**Table 3**). The radiomics signature also showed a high predictive value for CS-PCa in the test and validation sets (**Table 4**). The regression formula was as follows: prediction probability=−10.943+9.527*RS+1.742*PI-RADS. **Figure 3** shows the monogram.

**Table 3:** Univariate and multivariate logistic regression analyses in the training set.

	Univariable analyses		Multivariable analyses	
	OR (95% CI)	*P* value	OR (95% CI)	*P* value
Age (year)	0.967 (0.933, 1.003)	0.068	/	/
BMI (kg/m^2^)	0.891 (0.778, 1.021)	0.097	**/**	**/**
PSA	1.172 (1.080, 1.271)	**<0.001**	1.391 (0.991, 1.952)	0.056
Location	1.616 (0.950, 2.751)	0.077	/	/
PI-RADS	7.120 (3.569, 14.202)	**<0.001**	7.688 (1.594, 37.085)	**0.011**
Radiomics signature	4.517×10^4^ (899.309, 2268910.875)	**<0.001**	7.650×10^5^ (128.450, 4.560×10^9^)	**0.002**

OR, odds ratio.

Bold values mean p<0.05.

**Table 4:** Multivariate logistic regression analysis in the test and validation sets.

	Test set (n=60)		Validation set 1 (n=66)		Validation set 2 (n=122)	
	OR (95% CI)	*P* value	OR (95% CI)	*P* value	OR (95% CI)	*P* value
PSA	1.155 (0.904, 1.475)	0.250	1.185 (0.941, 1.492)	0.150	1.001 (0.996, 1.007)	0.627
PI-RADS	14.204 (1.150, 175.495)	**0.039**	4.751 (0.916, 24.655)	0.064	4.065 (1.833, 9.017)	**0.001**
Radiomics signature	9.420×10^6^ (1.206, 7.351×10^13^)	**0.047**	11624.241 (6.780, 1.993×10^7^)	**0.014**	1.021 (1.011, 1.031)	**<0.001**

OR, odds ratio.

Bold values mean p<0.05.

**Figure 4:**
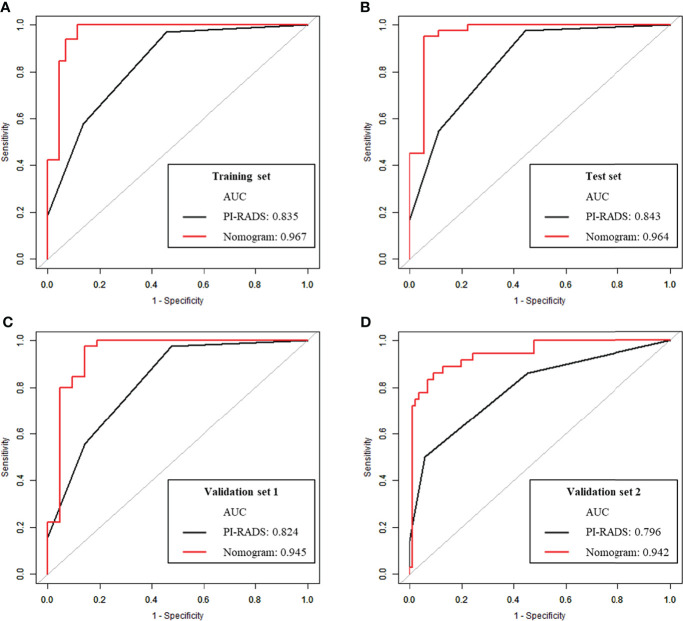
ROC curve analysis of the nomogram and PI-RADS for CS-PCa prediction. **(A)** In the training set. **(B)** In the test set. **(C)** In validation set 1. **(D)** In validation set 2.

**Figure 5:**
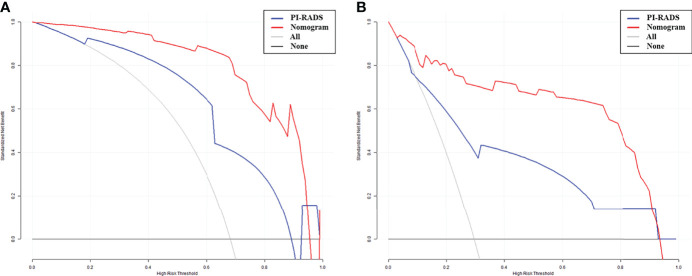
Decision curve analysis (DCA) of the nomogram and PI-RADS models. X-axis, risk threshold of CS-PCa; Y-axis, net benefit. Black line, all cases assumed to be clinically insignificant; gray line, all cases considered clinically significant. The nomogram model had enhanced net benefit compared with the PI-RADS at large probability thresholds (0.0-0.9). **(A)** In validation set 1. **(B)** In validation set 2.

AUCs for the nomogram were 0.967, 0.964, 0.945 and 0.942 in the training set, test set, validation set 1 and validation set 2, respectively. The Hosmer-Lemeshow test revealed the nomogram model had favorable calibration in all cohorts (*p*>0.05); details are listed in **Supplementary Table 3**. In all data sets, the nomogram showed elevated AUCs in comparison with the PI-RADS utilized alone. The DeLong test demonstrated significant differences (all *p*<0.05). NRIs were 0.326 to 0.372, showing the nomogram had an improved clinical utility compared with the PI-RADS for CS-PCa (**Table 5** and **Figure 4**). DCA of validation cohorts confirmed the nomogram’s superiority over the PI-RADS at large probability thresholds (**Figure 5**).

**Table 5:** ROC curve analysis and comparison of prediction models in all data sets.

		AUC	95% CI	Specificity	Sensitivity	Accuracy	*P* value	NRI
**Training set (n=141)**	PI-RADS	0.835	0.766-0.904	0.545	0.969	0.837	<0.001	0.372
	Nomogram	0.967	0.930-1.000	0.886	1.000	0.964		
**Test set (n=60)**	PI-RADS	0.843	0.737-0.948	0.556	0.976	0.850	0.01	0.365
	Nomogram	0.964	0.904-1.000	0.944	0.952	0.950		
**Validation set 1 (n=66)**	PI-RADS	0.824	0.719-0.929	0.524	0.978	0.833	0.01	0.333
	Nomogram	0.945	0.869-1.000	0.857	0.978	0.939		
**Validation set 2 (n=122)**	PI-RADS	0.796	0.710-0.882	0.942	0.500	0.812	<0.001	0.326
	Nomogram	0.942	0.896-0.987	0.907	0.861	0.893		

AUC, area under the curve; NRI, net reclassification index.

## Discussion

This work showed that whole prostate (T2WI) + lesion (DWI) was the best segmentation for radiomics model building. According to the AUC, NRI, and DCA results, a radiomics nomogram was developed, which seems to have higher predictive ability than the PI-RADS for CS-PCa in three hospital databases. Clinicians can use this model to more accurately screen patients with CS-PCa before surgery and conduct individuated treatments.

The European Association of Urology’ Guidelines on Prostate Cancer recommend active surveillance and follow-up observation for PCa patients with a Gleason score (GS) < 7, whereas clinically significant prostate cancer (CS-PCa) patients with GS ≥ 7 should undergo timely treatment and intervention because of increased risk of disease progression and short overall survival (2). Therefore, accurate clinical assessment is vital for patients to choose the best treatment.

In recent years, multiparametric MRI has been increasingly utilized for PCa’s qualitative evaluation (19, 20). The Prostate Imaging Reporting and Data System (PI-RADS) was proposed for better standardization of prostate MRI performance and image interpretation. PI-RADS guidelines v2.1 in 2019 introduced the concept of biparametric magnetic resonance imaging (including T2WI and DWI only) to simplify prostate MRI (21). Prostate MRI categorizes suspected PCa into low- and high risk types, considering risk scores from 1 to 5. PI-RADS grades of 3-5 are recommended to undergo MRI-directed biopsy (22), which could decrease the amounts of avoidable biopsies. However, such approach may miss a small portion of CS-PCas (23), due to low cancer detection rates, i.e., only 6% (0-20%) and 9% (5-13%) for PI-RADS 1 and PI-RADS 2, respectively, in patient level analysis (4). In addition, the commonly used clinical application of the PSA shows limitations, including overdiagnosis and resulting overtreatment (24, 25). Therefore, novel methods for timely and accurate PCa risk stratification are urgently required for improving patient prognosis.

Radiomics is a novel approach that converts traditional medical imaging findings into data mining and high-throughput quantitative analysis. The analysis approach of radiomics provides a non-invasive tool for evaluating the biological characteristics and heterogeneity of prostate cancer more comprehensively and quantitatively than morphological visual representation. Several studies have demonstrated that the current MRI-related radiomics application could be widely used for GS assessment in PCa (14–17). Although they found that multiparametric radiomics models show great potential in predicting GS, there is currently no comparative assessment of different combinations of sequences and patterns of segmentation for model building, which can yield higher clinical benefit for CS-PCa with external validation.

The most valuable aspect of the present study is the multi-pattern approach that enhances MRI-based radiomics by mining complementary information provided by multi-pattern MRI and considering the heterogeneity of tumors for predicting differential features involved in CS-PCa (26). Among the factors that affecting radiomics assessment, segmentation represents the first critical step of imaging processing. Manual ROI drawing represents the most conventionally utilized segmentation method nowadays (27). Most prior studies assessed lesion-derived radiomics models with AUCs from 0.648 to 0.910 (14–16). Gong et al. (17) investigated the potential of prostate gland radiomic features in identifying GS, with an AUC of 0.794 in the validation cohort. However, the various patterns of segmentation for model building have been less discussed and requires further quantitative assessment. Therefore, in this study, we established multi-pattern segmentations, including prostate lesions (T2WI or DWI), whole prostate (T2WI or DWI), and the combination of different methods, which were applied for radiomics analysis to detect clinically significant prostate cancer. Following feature selection, 10 optimal features based on the whole prostate (T2WI) + lesion (DWI) model were selected to develop a radiomics signature for preoperative prediction of CS-PCa, with favorable discriminatory potential (**Table 2**). A possible explanation is that the whole prostate (T2WI) model contained phenotypic features for the entire prostate, while the lesion (DWI) model involved heterogeneous data describing microcirculation for the focal lesion.

Since the PI-RADS v2.1 introduced the biparametric prostate MRI, which was widely recognized by radiologists and urologists, several prior studies extracted radiomic features from T2W and DWI (14, 16, 28, 29). Thus, combining biparametric MRI and deep mining of correlations among distinct radiomics features could allow a comprehensive assessment of tumor heterogeneity, which might increase the predictive efficiency and potentially guide in distinguishing cases requiring individualized treatments (30–32).

The second noteworthy aspect of the current study is that the radiomics signature and PI-RADS were combined to develop a radiomics nomogram with improved discriminatory ability, which constitutes a visualization tool to predict CS-PCa. Zhang et al. reported a radiomics nomogram model, which did not incorporate the PI-RADS v2 score, showed an AUC of 0.910 (15). Montoya et al. reported that the use of radiomics model failed to outperform PI-RADS v2.1 scales and their combination did not lead to further performance gains (AUC=0.830, *p*>0.05) (28). However, our results showed that the nomogram model incorporated subjective evaluation exhibited a higher AUC compared with the PI-RADS alone (*p*<0.05) in all cohorts. NRI analysis determined the predictive value was improved by using the nomogram in lieu of the traditional PI-RADS v2.1, and good clinical usefulness was demonstrated by DCA. These data suggest the developed nomogram could be utilized to guide clinical practice.

The third vital aspect of this study is that we had two actual external validation datasets, adding value to our previous reports. Using external cohorts is very helpful for overcoming the weakness that the developed model has no exposure to a validation cohort in the training phase.

However, the current study still had some limitations. First, an important drawback of the current retrospective trial was its relatively small sample size. This implies selection bias and low generalizability of the obtained results, although external validation cohorts were analyzed. Therefore, larger multicenter studies are warranted for reducing the effects of selection bias on model accuracy. Secondly, the imaging segmentation approach was manual rather than semi-automatic/automatic delineation, favoring subjective errors, with no suitability for large data processing (33). Thirdly, the current work failed to develop and validate deep learning tools for the prediction of CS-PCa, which may show more advantages and deserve further investigation (34).

## Conclusion

Overall, based on preoperative biparametric MRI [whole prostate (T2WI) + lesion (DWI)], a quantitative radiomics signature was built. The nomogram model combined with the radiomics signature and PI-RADS had improved clinical benefit in comparison with the subjective evaluation only in predicting clinically significant prostate cancer.

## Data availability statement

The original contributions presented in the study are included in the article/**Supplementary Material**. Further inquiries can be directed to the corresponding authors.

## Ethics statement

Written informed consent was not obtained from the individual(s) for the publication of any potentially identifiable images or data included in this article.

## Author contributions

JL, CS, YL, and FS conceived the project. XM acquired the data. PX and GJ analyzed and interpreted the patient data regarding radiomics features. ZL and HL performed statistical analyses and feature extraction. ZL, PX, and GJ was a major contributor in writing the manuscript. All authors contributed to the article and approved the submitted version.

## Funding

The present study was supported by the Project of the Action Plan of Major Diseases Prevention and Treatment (2017ZX01001-S12), the Special Project of Integrated Traditional Chinese and Western Medicine in General Hospitals of Shanghai (ZHYY-ZXYJHZX-201901), and the Changhai hospital discipline construction project (2020YXK034). The funders developed the main idea and designed the study.

## Conflict of interest

The authors declare that the research was conducted in the absence of any commercial or financial relationships that could be construed as a potential conflict of interest.

## Publisher’s note

All claims expressed in this article are solely those of the authors and do not necessarily represent those of their affiliated organizations, or those of the publisher, the editors and the reviewers. Any product that may be evaluated in this article, or claim that may be made by its manufacturer, is not guaranteed or endorsed by the publisher.

## Supplementary material

Supplementary Table 1 Main MRI sequences and parameters.

Supplementary Table 2 Description of the selected radiomics features with associated feature groups and filters.

Supplementary Table 3 Prediction performances of the nomogram model in all data sets.

Supplementary Figure 1 Repeatability of radiomics features based on ICCs in different cohorts.

Supplementary Figure 2 ROC curves for 10 models, PSA and PI-RADS, and comparisons (Delong test).

Supplementary Figure 3 Correlation analysis of the selected ten optimal features of the whole prostate (T2WI) + lesion (DWI) model.

## References

[1] Culp MB Soerjomataram I Efstathiou JA Bray F Jemal A. Recent global patterns in prostate cancer incidence and mortality rates. Eur Urol (2020) 77(1):38–52. doi: 10.1016/j.eururo.2019.08.005 31493960

[2] Mottet N van den Bergh RCN Briers E Van den Broeck T Cumberbatch MG De Santis M. EAU-EANM-ESTRO-ESUR-SIOG guidelines on prostate cancer-2020 update. part 1: Screening, diagnosis, and local treatment with curative intent. Eur Urol (2021) 79(2):243–62.doi: 10.1016/j.eururo.2020.09.042 33172724

[3] Goel S Shoag JE Gross MD Al Hussein Al Awamlh B Robinson B. Concordance between biopsy and radical prostatectomy pathology in the era of targeted biopsy: a systematic review and meta-analysis. Eur Urol Oncol (2020) 3(1):10–20. doi: 10.1016/j.euo.2019.08.001. 31492650

[4] Goel S Shoag JE Gross MD. Concordance between biopsy and radical prostatectomy pathology in the era of targeted biopsy: a systematic review and meta-analysis. Eur Urol Oncol (2020) 3(1):10–20. doi: 10.1016/j.euo.2019.08.001 31492650

[5] Oerther B Engel H Bamberg F Sigle A Gratzke C Benndorf M. Cancer detection rates of the PI-RADSv2.1 assessment categories: systematic review and meta-analysis on lesion level and patient level. Prostate Cancer Prostatic Dis (2021). doi: 10.1038/s41391-021-00417-1 PMC9184264 34230616

[6] Eldred-Evans D Burak P Connor MJ Day E Evans M Fiorentino F. Population-based prostate cancer screening with magnetic resonance imaging or ultrasonography: the IP1-PROSTAGRAM study. JAMA Oncol (2021) 7(3):395–402. doi: 10.1001/jamaoncol.2020.7456 33570542 PMC7879388

[7] Lambin P Rios-Velazquez E Leijenaar R Carvalho S van Stiphout RG Granton P. Radiomics: extracting more information from medical images using advanced feature analysis. Eur J Cancer (2012) 48(4):441–6. doi: 10.1016/j.ejca.2011.11.036 PMC4533986 22257792

[8] Kumar V Gu Y Basu S Berglund A Eschrich SA Schabath MB. Radiomics: the process and the challenges. Magn Reson Imaging (2012) 30(9):1234–48. doi: 10.1016/j.mri.2012.06.010 PMC3563280 22898692

[9] Aerts HJ Velazquez ER Leijenaar RT Parmar C Grossmann P Carvalho S. Decoding tumour phenotype by noninvasive imaging using a quantitative radiomics approach. Nat Commun (2014) 5:4006. doi: 10.1038/ncomms5006 24892406 PMC4059926

[10] Gillies RJ Kinahan PE Hricak H. Radiomics: images are more than pictures, they are data. Radiology (2016) 278:563–77. doi: 10.1148/radiol.2015151169 PMC4734157 26579733

[11] Li Z Li S Zang S Ma X Chen F Xia Y. Predicting treatment response to neoadjuvant chemoradiotherapy in rectal mucinous adenocarcinoma using an MRI-based radiomics nomogram. Front Oncol (2021) 11:671636. doi: 10.3389/fonc.2021.671636 34109121 PMC8181148

[12] Ma X Shen F Jia Y Xia Y Li Q Lu J. MRI-Based radiomics of rectal cancer: preoperative assessment of the pathological features. BMC Med Imaging (2019) 19(1):86. doi: 10.1186/s12880-019-0392-7 31747902 PMC6864926

[13] Liu M Ma X Shen F Xia Y Jia Y Lu J. MRI-Based radiomics nomogram to predict synchronous liver metastasis in primary rectal cancer patients. Cancer Med (2020) 9(14):5155–63. doi: 10.1002/cam4.3185 PMC7367643 32476295

[14] Toivonen J Montoya Perez I Movahedi P Merisaari H Pesola M Taimen P. Radiomics and machine learning of multisequence multiparametric prostate MRI: Towards improved non-invasive prostate cancer characterization. PloS One (2019) 14(7):e0217702. doi: 10.1371/journal.pone.0217702 31283771 PMC6613688

[15] Zhang GM Han YQ Wei JW Qi YF Gu DS Lei J. Radiomics based on MRI as a biomarker to guide therapy by predicting upgrading of prostate cancer from biopsy to radical prostatectomy. J Magn Reson Imaging (2020) 52(4):1239–48. doi: 10.1002/jmri.27138 32181985

[16] Chaddad A Kucharczyk MJ Niazi T. Multimodal radiomic features for the predicting gleason score of prostate cancer. Cancers (Basel) (2018) 10(8):249. doi: 10.3390/cancers10080249 PMC6116195 30060575

[17] Gong L Xu M Fang M He B Li H Fang X. The potential of prostate gland radiomic features in identifying the gleason score. Comput Biol Med (2022) 144:105318. doi: 10.1016/j.compbiomed.2022.105318 35245698

[18] Zwanenburg A Vallières M Abdalah MA Aerts HJWL Andrearczyk V Apte A. The image biomarker standardization initiative: standardized quantitative radiomics for high-throughput image-based phenotyping. Radiology (2020) 295(2):328–38. doi: 10.1148/radiol.2020191145 PMC7193906 32154773

[19] Ueno Y Tamada T Bist V Reinhold C Miyake H Tanaka U. Multiparametric magnetic resonance imaging: current role in prostate cancer management. Int J Urol (2016) 23(7):550–7. doi: 10.1111/iju.13119 27184019

[20] Aydın H Kızılgöz V Tekin BO. Overview of current multiparametric magnetic resonance imaging approach in the diagnosis and staging of prostate cancer. Kaohsiung J Med Sci (2015) 31(4):167–78. doi: 10.1016/j.kjms.2015.01.002 PMC11916159 25835272

[21] Turkbey B Rosenkrantz AB Haider MA Padhani AR Villeirs G Macura KJ. Prostate imaging reporting and data system version 2.1: 2019 update of prostate imaging reporting and data system version 2. Eur Urol (2019) 76(3):340–51. doi: 10.1016/j.eururo.2019.02.033 30898406

[22] Padhani AR Barentsz J Villeirs G Rosenkrantz AB Margolis DJ Turkbey B. PI-RADS steering committee: the PI-RADS multiparametric MRI and MRI-directed biopsy pathway. Radiology (2019) 292(2):464–74. doi: 10.1148/radiol.2019182946 PMC6677282 31184561

[23] Schoots IG Padhani AR Rouvière O Barentsz JO Richenberg J. Analysis of magnetic resonance imaging-directed biopsy strategies for changing the paradigm of prostate cancer diagnosis. Eur Urol Oncol (2020) 3(1):32–41. doi: 10.1016/j.euo.2019.10.001 31706946

[24] Salami SS Vira MA Turkbey B khoury M Yaskiv O Villani R. Multiparametric magnetic resonance imaging outperforms the prostate cancer prevention trial risk calculator in predicting clinically significant prostate cancer. Cancer (2014) 120(18):2876–82. doi: 10.1002/cncr.28790 24917122

[25] Bhat NR Vetter JM Andriole GL Shetty AS Ippolito JE Kim EH. Magnetic resonance imaging-defined prostate-specific antigen density significantly improves the risk prediction for clinically significant prostate cancer on biopsy. Urology (2019) 126:152–7. doi: 10.1016/j.urology.2018.12.010 30580005

[26] Li Z Dai H Liu Y Pan F Yang Y Zhang M. Radiomics analysis of multi-sequence MR images for predicting microsatellite instability status preoperatively in rectal cancer. Front Oncol (2021) 11:697497. doi: 10.3389/fonc.2021.697497 34307164 PMC8293900

[27] Rizzo S Botta F Raimondi S Origgi D Fanciullo C Morganti AG. Radiomics: the facts and the challenges of image analysis. Eur Radiol Exp (2018) 2(1):36. doi: 10.1186/s41747-018-0068-z 30426318 PMC6234198

[28] Montoya Perez I Merisaari H Jambor I Ettala O Taimen P Knaapila J. Detection of prostate cancer using biparametric prostate MRI, radiomics, and kallikreins: a retrospective multicenter study of men with a clinical suspicion of prostate cancer. J Magn Reson Imaging (2022) 55(2):465–77. doi: 10.1002/jmri.27811 34227169

[29] Zhang L Jiang D Chen C Yang X Lei H Kang Z. Development and validation of a multiparametric MRI-based radiomics signature for distinguishing between indolent and aggressive prostate cancer. Br J Radiol (2022) 95(1131):20210191. doi: 10.1259/bjr.20210191 34289319 PMC8978240

[30] Scialpi M. Simplified PI-RADS-based biparametric MRI: a rationale for detecting and managing prostate cancer. Clin Imaging (2021) 80:290–1. doi: 10.1016/j.clinimag.2021.07.024 34455239

[31] Zhang L Zhe X Tang M Zhang J Ren J Zhang X. Predicting the grade of prostate cancer based on a biparametric MRI radiomics signature. Contrast Media Mol Imaging (2021) 2021:7830909. doi: 10.1155/2021/7830909 35024015 PMC8718299

[32] Xu L Zhang G Shi B Liu Y Zou T Yan W. Comparison of biparametric and multiparametric MRI in the diagnosis of prostate cancer. Cancer Imaging (2019) 19(1):90. doi: 10.1186/s40644-019-0274-9 31864408 PMC6925429

[33] Price WN 2nd Cohen IG. Privacy in the age of medical big data. Nat Med (2019) 25:37–43. doi: 10.1038/s41591-018-0272-7 30617331 PMC6376961

[34] Hosny A Parmar C Quackenbush J Schwartz LH Aerts H. Artificial intelligence in radiology. Nat Rev Cancer (2018) 18:500–10. doi: 10.1038/s41568-018-0016-5 PMC6268174 29777175

