# Supplementary material for: Prediction of clinically significant prostate cancer with a multimodal MRI-based radiomics nomogram
Source: Front Oncol. 2022 Jul 15;12:918830. doi: 10.3389/fonc.2022.918830 (PMC9334707; doi:10.3389/fonc.2022.918830)
Supplement: Supplementary Table 1 — Main MRI sequences and parameters. [file Table_1.docx]

**Supplemental Table 1.** **Main MRI sequences protocol and parameters**

| **Cohort** | **Cohort 1** | **Cohort 2** | **Cohort 3** |
| --- | --- | --- | --- |
| **Scanner** | **GE**  **Discovery 750w** | **United Imaging**  **uMR 890** | **GE**  **Signa HDX** |
| **Magnetic field strength** | 3.0T | 3.0T | 3.0T |
| **Axial T2WI** |  |  |  |
| Field of view (mm) | 200×200 | 200×200 | 240×240 |
| Section thickness (mm) | 4 | 3.5 | 4 |
| Matrix | 352×352 | 304×304 | 320×224 |
| TR/TE (ms) | 5654/118 | 5118/126 | 4600/120 |
| Bandwidth (kHz) | 62.5 | 240 | 31.25 |
| Flip angle (°) | 110 | 90 | 90 |
| **Axial DWI** |  |  |  |
| Field of view (mm) | 320×320 | 200×200 | 300×300 |
| Section thickness (mm) | 4 | 3.5 | 4 |
| Matrix | 128×128 | 112×112 | 130×96 |
| TR/TE (ms) | 2451/Minimum | 3000/58 | 6475/73 |
| b values (s/mm^2^) | 500, 1000, 1500, 2000, 3000 | 50, 800, 1500 | 0, 1500 |

TR/TE: Repetition time/echo time.

Cohort 1: Changhai Hospital; Cohort 2: Ruijin Hospital Luwan Branch; Cohort 3: 989th Hospital of the joint logistic support force of the Chinese People's Liberation Army.
