# Supplementary material for: Prediction of clinically significant prostate cancer with a multimodal MRI-based radiomics nomogram
Source: Front Oncol. 2022 Jul 15;12:918830. doi: 10.3389/fonc.2022.918830 (PMC9334707; doi:10.3389/fonc.2022.918830)
Supplement: Supplementary Table 2 — Description of the selected radiomics features with associated feature groups and filters. [file Table_2.docx]

**Supplemental Table 2. Description of the selected radiomics features with associated feature groups and filters**

| **Model** | **Radiomics feature** | **Radiomics class** | **Filter** |
| --- | --- | --- | --- |
| **DWI (lesion + whole prostate)** | ZoneEntropy | GLSZM | DWI-l_wavelet-LHL |
|  | JointEntropy | GLCM | DWI-l_wavelet-LLL |
|  | RunVariance | GLRLM | DWI-l_wavelet-LLL |
|  | Kurtosis | first order | DWI-l_squareroot |
|  | HighGrayLevelRunEmphasis | GLRLM | DWI-w_original |
|  | HighGrayLevelRunEmphasis | GLRLM | DWI-w_logarithm |
|  | HighGrayLevelRunEmphasis | GLRLM | DWI-w_squareroot |
|  | HighGrayLevelZoneEmphasis | GLSZM | DWI-l_original |
|  | HighGrayLevelZoneEmphasis | GLSZM | DWI-l_logarithm |
|  | SizeZoneNonUniformity | GLSZM | DWI-w_wavelet-HHH |
|  | ZoneEntropy | GLSZM | DWI-w_original |
|  | ZoneEntropy | GLSZM | DWI-w_logarithm |
|  | ZoneEntropy | GLSZM | DWI-w_squareroot |
|  | Kurtosis | first order | DWI-w_squareroot |
| **DWI (lesion)** | Busyness | NGTDM | wavelet-HLH |
|  | JointEntropy | GLCM | wavelet-LLL |
|  | LargeAreaLowGrayLevelEmphasis | GLSZM | wavelet-HLH |
|  | ZoneVariance | GLSZM | wavelet-HLH |
|  | RunLengthNonUniformity | GLRLM | original |
|  | RunLengthNonUniformity | GLRLM | logarithm |
|  | LargeDependenceLowGrayLevelEmphasis | GLDM | wavelet-LHH |
|  | 10Percentile | first order | wavelet-LLH |
|  | ZoneVariance | GLSZM | wavelet-LLH |
|  | ZoneEntropy | GLSZM | wavelet-LLL |
| **DWI (whole prostate)** | LargeAreaEmphasis | GLSZM | wavelet-LLL |
|  | LargeAreaHighGrayLevelEmphasis | GLSZM | wavelet-LLL |
|  | RootMeanSquared | first order | exponential |
|  | SizeZoneNonUniformity | GLSZM | wavelet-HHH |
|  | Kurtosis | first order | squareroot |
| **Whole prostate (DWI) + lesion (T2WI)** | LargeDependenceHighGrayLevelEmphasis | GLDM | T2WI-l_wavelet-LHH |
|  | SizeZoneNonUniformity | GLSZM | T2WI-l_original |
|  | SizeZoneNonUniformity | GLSZM | T2WI-l_logarithm |
|  | SizeZoneNonUniformity | GLSZM | T2WI-l_squareroot |
|  | LargeAreaEmphasis | GLSZM | DWI-w_wavelet-LLL |
|  | Variance | first order | T2WI-l_lbp-2D |
|  | LargeAreaHighGrayLevelEmphasis | GLSZM | DWI-w_wavelet-LLL |
|  | Busyness | NGTDM | T2WI-l_original |
|  | Busyness | NGTDM | T2WI-l_logarithm |
|  | Busyness | NGTDM | T2WI-l_squareroot |
|  | Median | first order | T2WI-l_lbp-2D |
|  | TotalEnergy | first order | DWI-w_exponential |
| **Lesion (DWI + T2WI)** | SizeZoneNonUniformity | GLSZM | T2WI-l_original |
|  | SizeZoneNonUniformity | GLSZM | T2WI-l_logarithm |
|  | LargeAreaLowGrayLevelEmphasis | GLSZM | T2WI-l_wavelet-LHL |
|  | GrayLevelNonUniformity | GLSZM | T2WI-l_wavelet-LHH |
|  | LargeDependenceHighGrayLevelEmphasis | GLDM | T2WI-l_wavelet-LLL |
|  | RunLengthNonUniformity | GLRLM | T2WI-l_wavelet-LLL |
|  | 10Percentile | first order | DWI-l_wavelet-LLH |
|  | ZoneEntropy | GLSZM | DWI-l_wavelet-LLL |
| **T2WI (lesion + whole prostate)** | SizeZoneNonUniformity | GLSZM | T2WI-l_original |
|  | HighGrayLevelZoneEmphasis | GLSZM | T2WI-w_wavelet-LLL |
|  | LargeDependenceEmphasis | GLDM | T2WI-l_wavelet-LHH |
|  | Busyness | NGTDM | T2WI-l_original |
|  | Busyness | NGTDM | T2WI-l_logarithm |
|  | Busyness | NGTDM | T2WI-l_squareroot |
|  | LongRunEmphasis | GLRLM | T2WI-l_wavelet-LHH |
|  | Energy | first order | T2WI-w_squareroot |
|  | LongRunHighGrayLevelEmphasis | GLRLM | T2WI-l_wavelet-LHL |
|  | Median | first order | T2WI-l_lbp-2D |
|  | LargeAreaHighGrayLevelEmphasis | GLSZM | T2WI-w_original |
|  | LargeAreaHighGrayLevelEmphasis | GLSZM | T2WI-w_logarithm |
|  | LargeAreaHighGrayLevelEmphasis | GLSZM | T2WI-w_squareroot |
|  | DependenceEntropy | GLDM | T2WI-l_wavelet-LLL |
|  | Skewness | first order | T2WI-l_wavelet-LHH |
|  | LongRunHighGrayLevelEmphasis | GLRLM | T2WI-w_wavelet-HLH |
|  | TotalEnergy | first order | T2WI-l_wavelet-LLH |
|  | Busyness | NGTDM | T2WI-l_wavelet-LLL |
|  | LargeAreaHighGrayLevelEmphasis | GLSZM | T2WI-w_wavelet-LLL |
| **T2WI (lesion)** | LargeDependenceLowGrayLevelEmphasis | GLDM | original |
|  | LargeDependenceLowGrayLevelEmphasis | GLDM | logarithm |
|  | LargeDependenceLowGrayLevelEmphasis | GLDM | squareroot |
|  | SizeZoneNonUniformity | GLSZM | original |
|  | SizeZoneNonUniformity | GLSZM | logarithm |
|  | SizeZoneNonUniformity | GLSZM | squareroot |
|  | Busyness | NGTDM | original |
|  | Busyness | NGTDM | logarithm |
|  | Busyness | NGTDM | squareroot |
|  | Energy | first order | wavelet-HLL |
|  | Skewness | first order | square |
|  | SizeZoneNonUniformity | GLSZM | wavelet-HLH |
| **T2WI (whole prostate)** | HighGrayLevelZoneEmphasis | GLSZM | wavelet-LLL |
|  | LongRunLowGrayLevelEmphasis | GLRLM | wavelet-HLH |
|  | SmallAreaHighGrayLevelEmphasis | GLSZM | wavelet-LLL |
|  | LongRunHighGrayLevelEmphasis | GLRLM | wavelet-HHL |
| **Whole prostate (T2WI) + lesion (DWI)** | RunVariance | GLRLM | DWI-l_wavelet-LLL |
|  | HighGrayLevelZoneEmphasis | GLSZM | T2WI-w_wavelet-LLL |
|  | ZoneEntropy | GLSZM | DWI-l_wavelet-LHL |
|  | HighGrayLevelRunEmphasis | GLRLM | DWI-l_original |
|  | HighGrayLevelRunEmphasis | GLRLM | DWI-l_logarithm |
|  | HighGrayLevelRunEmphasis | GLRLM | DWI-l_squareroot |
|  | RunLengthNonUniformity | GLRLM | DWI-l_wavelet-LLL |
|  | LongRunEmphasis | GLRLM | T2WI-w_wavelet-HLH |
|  | SmallAreaHighGrayLevelEmphasis | GLSZM | T2WI-w_wavelet-LLL |
|  | LongRunHighGrayLevelEmphasis | GLRLM | T2WI-w_wavelet-HHL |
| **Whole prostate (DWI + T2WI)** | HighGrayLevelZoneEmphasis | GLSZM | T2WI-w_wavelet-LLL |
|  | RootMeanSquared | first order | DWI-w_exponential |
|  | HighGrayLevelRunEmphasis | GLRLM | DWI-w_original |
|  | SizeZoneNonUniformity | GLSZM | DWI-w_wavelet-HHH |
|  | LongRunHighGrayLevelEmphasis | GLRLM | T2WI-w_wavelet-HLH |
|  | LongRunLowGrayLevelEmphasis | GLRLM | DWI-w_wavelet-LLL |

GLSZM = Gray level size zone matrix.

GLDM = Gray Level dependence.

GLRLM = Gray level run length matrix.

NGTDM = Neighborhood gray tone difference matrix.

Wavelet: The wavelet transform decomposes the tumor area image into low-frequency components (L) or high-frequency components (H) in the x, y, and z axes.
