# Supplementary material for: Prediction of clinically significant prostate cancer with a multimodal MRI-based radiomics nomogram
Source: Front Oncol. 2022 Jul 15;12:918830. doi: 10.3389/fonc.2022.918830 (PMC9334707; doi:10.3389/fonc.2022.918830)
Supplement: Supplementary Table 3 — Prediction performances of the nomogram model in all data sets. [file Table_3.docx]

**Supplemental Table 3. Prediction performances of the nomogram model in all data sets**

|  | **AUC** | **95% CI** | **Specificity** | **Sensitivity** | **Accuracy** | **PLR** | **NLR** | **PPV** | **NPV** | **Hosmer & Lemeshow test** |
| --- | --- | --- | --- | --- | --- | --- | --- | --- | --- | --- |
| **Training set (n=141)** | 0.967 | 0.930-1.000 | 0.886 | 1.000 | 0.964 | 8.800 | 0.000 | 0.951 | 1.000 | 0.129 |
| **Test set (n=60)** | 0.964 | 0.904-1.000 | 0.944 | 0.952 | 0.950 | 17.143 | 0.050 | 0.976 | 0.895 | 0.099 |
| **Validation set 1 (n=66)** | 0.945 | 0.869-1.000 | 0.857 | 0.978 | 0.939 | 6.844 | 0.026 | 0.936 | 0.947 | 0.292 |
| **Validation set 2 (n=122)** | 0.942 | 0.896-0.987 | 0.907 | 0.861 | 0.893 | 9.257 | 0.153 | 0.795 | 0.940 | 0.167 |

AUC: area under the curve; PLR, positive likelihood ratio; NLR, negative likelihood ratio; NPV, negative predictive value; PPV, positive predictive value.
