# Supplementary figures and images for: Prediction of clinically significant prostate cancer with a multimodal MRI-based radiomics nomogram
Source: Front Oncol. 2022 Jul 15;12:918830. doi: 10.3389/fonc.2022.918830 (PMC9334707; doi:10.3389/fonc.2022.918830)

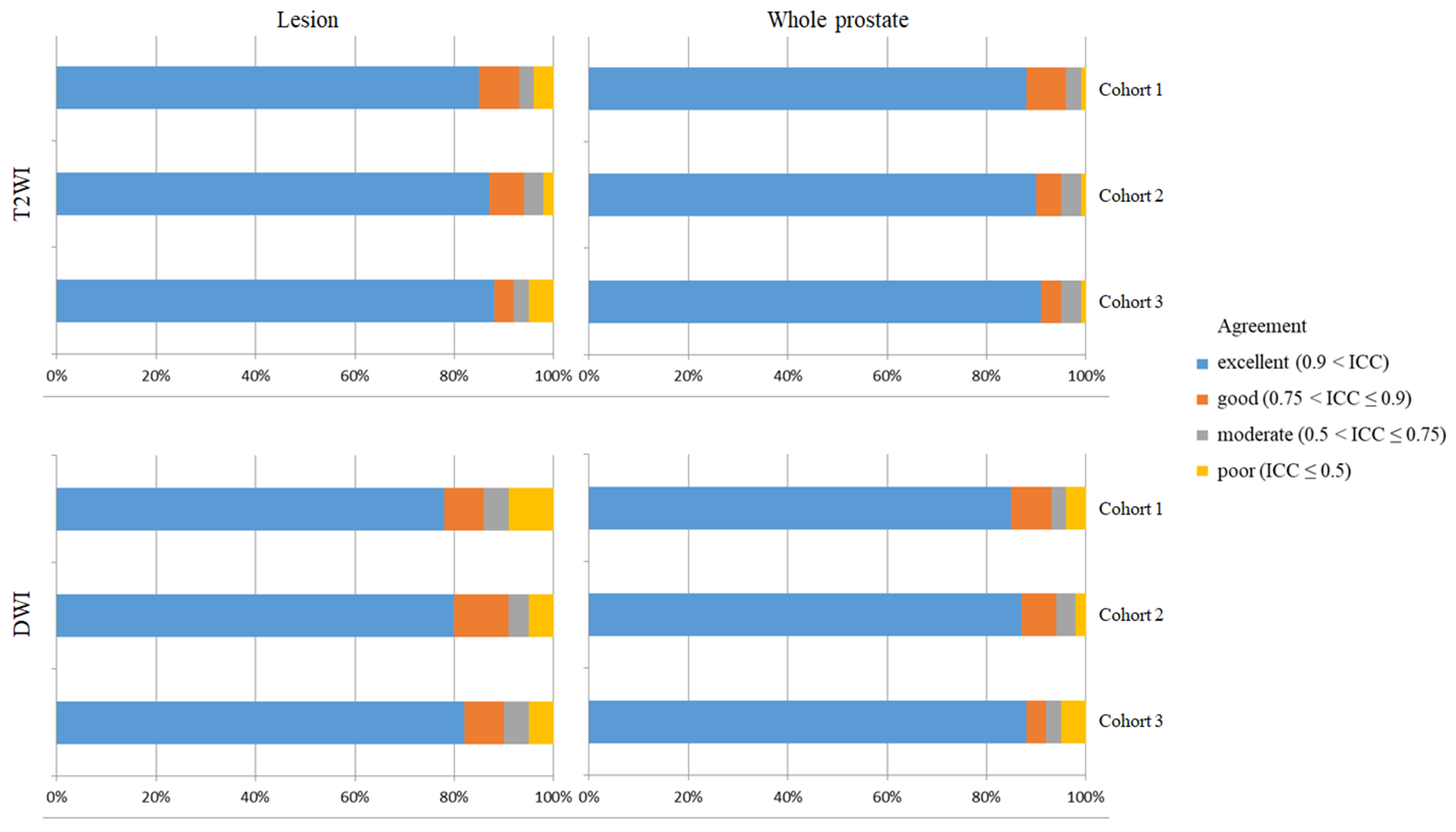

Supplement: Supplementary Figure 1 — Repeatability of radiomics features based on ICCs in different cohorts. [file Image_1.tif]

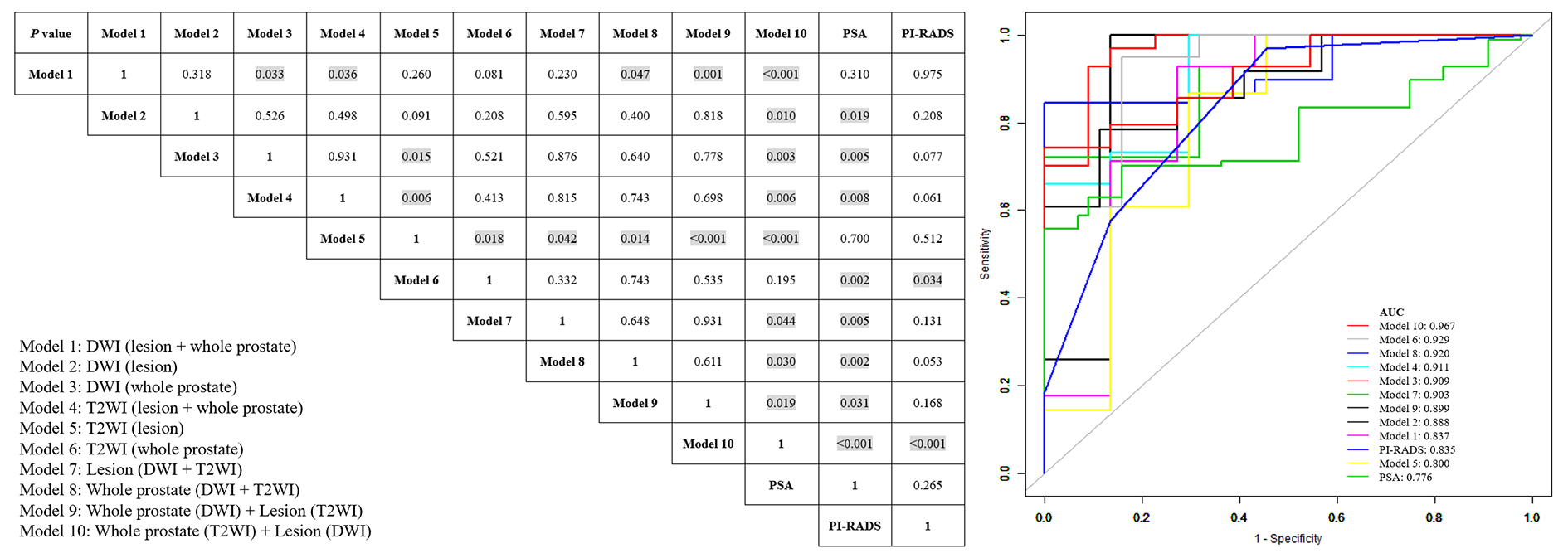

Supplement: Supplementary Figure 2 — ROC curves for 10 models, PSA and PI-RADS, and comparisons (Delong test). [file Image_2.tif]

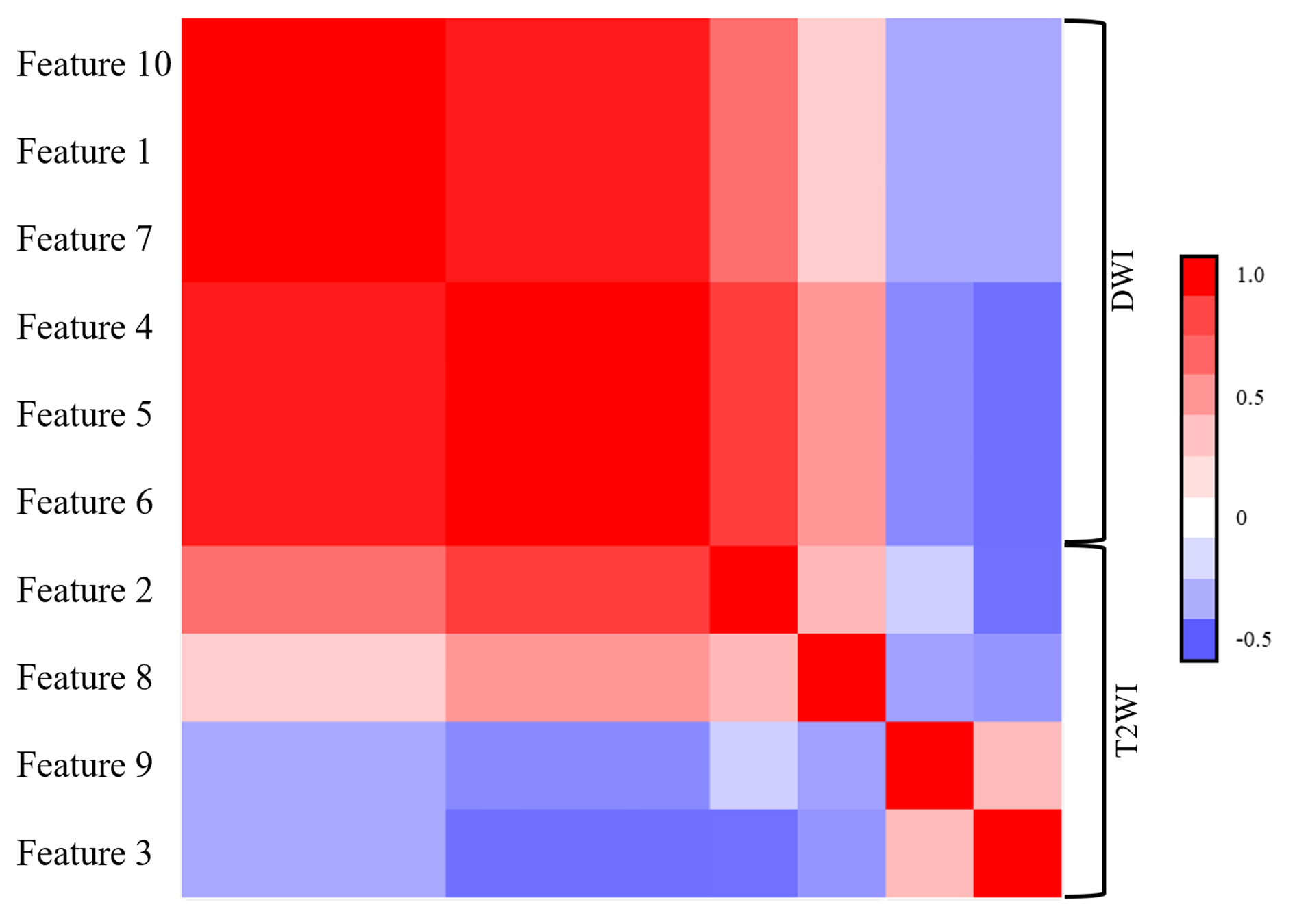

Supplement: Supplementary Figure 3 — Correlation analysis of the selected ten optimal features of the whole prostate (T2WI) + lesion (DWI) model. [file Image_3.tif]
